# MRI and CT imaging for preoperative target volume delineation in breast-conserving therapy

**DOI:** 10.1186/1748-717X-9-63

**Published:** 2014-02-26

**Authors:** Mariska D den Hartogh, Marielle EP Philippens, Iris E van Dam, Catharina E Kleynen, Robbert JHA Tersteeg, Ruud M Pijnappel, Alexis NTJ Kotte, Helena M Verkooijen, Maurice AAJ van den Bosch, Marco van Vulpen, Bram van Asselen, HJG Desirée van den Bongard

**Affiliations:** 1 Department of Radiotherapy, University Medical Center Utrecht, HP Q00.118, PO Box 85500, 3508 GA Utrecht, The Netherlands; 2 Department of Radiology, University Medical Center Utrecht, Utrecht, The Netherlands

**Keywords:** MRI, CT, Supine, Interobserver variability, Preoperative, GTV, Tumor bed, Breast cancer, Radiotherapy

## Abstract

**Background:**

Accurate tumor bed delineation after breast-conserving surgery is important. However, consistency among observers on standard postoperative radiotherapy planning CT is low and volumes can be large due to seroma formation. A preoperative delineation of the tumor might be more consistent. Therefore, the purpose of this study was to determine the consistency of preoperative target volume delineation on CT and MRI for breast-conserving radiotherapy.

**Methods:**

Tumors were delineated on preoperative contrast-enhanced (CE) CT and newly developed 3D CE-MR images, by four breast radiation oncologists. Clinical target volumes (CTVs) were created by addition of a 1.5 cm margin around the tumor, excluding skin and chest wall. Consistency in target volume delineation was expressed by the interobserver variability. Therefore, the conformity index (CI), center of mass distance (dCOM) and volumes were calculated. Tumor characteristics on CT and MRI were scored by an experienced breast radiologist.

**Results:**

Preoperative tumor delineation resulted in a high interobserver agreement with a high median CI for the CTV, for both CT (0.80) and MRI (0.84). The tumor was missed on CT in 2/14 patients (14%). Leaving these 2 patients out of the analysis, CI was higher on MRI compared to CT for the GTV (p < 0.001) while not for the CTV (CT (0.82) versus MRI (0.84), p = 0.123). The dCOM did not differ between CT and MRI. The median CTV was 48 cm^3^ (range 28–137 cm^3^) on CT and 59 cm^3^ (range 30–153 cm^3^) on MRI (p < 0.001). Tumor shapes and margins were rated as more irregular and spiculated on CE-MRI.

**Conclusions:**

This study showed that preoperative target volume delineation resulted in small target volumes with a high consistency among observers. MRI appeared to be necessary for tumor detection and the visualization of irregularities and spiculations. Regarding the tumor delineation itself, no clinically relevant differences in interobserver variability were observed. These results will be used to study the potential for future MRI-guided and neoadjuvant radiotherapy.

**Trial registration:**

International Clinical Trials Registry Platform NTR3198.

## Background

The standard treatment of early-stage breast cancer is lumpectomy, or wide local excision, followed by whole breast irradiation with an additional boost dose to the tumor bed (TB) in patients with a higher risk of local recurrence [1,2]. Since most local recurrences occur in or nearby the TB, several accelerated partial breast irradiation (APBI) studies are ongoing in early-stage breast cancer patients. APBI targets the breast tissue immediately surrounding the TB. The advantages of APBI are a shorter overall treatment time and a potential dose reduction in the normal tissues (i.e. breast, heart and lung) compared to whole breast irradiation [3]. Accurate TB delineation on the radiotherapy planning CT scan after lumpectomy is important for both TB boost irradiation and APBI. However, in radiotherapy practice, there is no gold standard to validate the accuracy of our target volume delineation after lumpectomy. As an alternative, consensus among observers is often used to assess the precision of our target volume delineation. The degree of consensus is generally called the interobserver variability (IOV), and quantified by a conformity index (CI) which is the volume of agreement among observers divided by the total encompassing volume. The current CT guided delineation after lumpectomy is prone to a high IOV. Several studies showed a low CI and a large distance between the centers of mass (dCOM) among observers [4-13].

Besides the high IOV in the current postoperative radiotherapy setting, there is also the concern of large postoperative treatment volumes due to seroma and hematoma formation. Irradiation of these disproportionally large target volumes can lead to extended subcutaneous fibrosis, poor cosmetic results and even missing the target [14-17]. Furthermore, these large volumes can cause low-risk patients aiming for APBI to be ineligible for this treatment due to the inability to meet the dose-volume constraints [18,19].

The poor consistency in target volume definition and large volumes after lumpectomy might be avoided by irradiating the tumor preoperatively. Since the tumor is still in situ without any seroma formation, this would probably lead to a high delineation precision and small treatment volumes. Several groups are studying the potential for neoadjuvant irradiation in early stage breast cancer patients [18,20,21]. In these studies, IOV and normal tissue dose were reduced, which shows that neoadjuvant irradiation could result in more precise target volume definition and localization and smaller volumes [20-22]. Furthermore, Bondiau et al. reported the feasibility of a neoadjuvant stereotactic body irradiation in combination with neoadjuvant chemotherapy in locally advanced breast cancer patients [23].

Alternatively, preoperative imaging in radiotherapy supine position might also have potential value to improve the standard post-lumpectomy TB delineation, since it provides additional information about the original tumor location [11].

To correctly delineate the tumor, imaging quality is of great importance. Since it is unknown what the optimal imaging modality for preoperative target volume delineation is, delineation was studied on both contrast-enhanced (CE) CT and MRI. In daily clinical practice, CT is the standard imaging modality for target volume delineation in breast cancer patients. However, MRI has a superior soft tissue contrast which can be explored with different sequences to show endogenous contrast or the distribution of an administered contrast agent. This enables differentiation between the tumor and benign lesions like post-biopsy hematomas or cysts. Furthermore, MRI has a high sensitivity for detection of invasive breast cancer and a good correlation with histopathology findings [24,25]. However, standard diagnostic MRI is performed in prone position, while patients in most departments are irradiated in supine position. Acquiring images in supine radiotherapy position is generally limited by narrow bore sizes of standard MRI scanners. Therefore a new MRI protocol was designed in a wide bore MRI scanner.

The purpose of this study was to quantify the consistency of preoperative target volume delineation for breast-conserving radiotherapy. To identify the best imaging modality for preoperative target volume delineation, preoperative delineation was performed on both CE-CT and a newly developed 3D CE-MRI in supine radiotherapy position.

## Methods

### Patients and selection

The study was approved by our institutional review board and registered in the International Clinical Trials Registry Platform (NTR3198). Fourteen early-staged breast cancer patients, scheduled for lumpectomy at the University Medical Center Utrecht or St. Antonius hospital, were included in this study. All patients gave written informed consent. Patients eligible for inclusion had a clinical T1-T2, N0 staged adenocarcinoma of the breast and were scheduled for lumpectomy and sentinel node procedure. Patients with lobular carcinoma, a history of ipsilateral breast surgery, contra-indications for 1.5 Tesla MRI, iodine allergy, and patients who received neoadjuvant treatment were not eligible. In case of additional suspected findings on study MRI or CT imaging, patients were referred to their physician for additional diagnostic work-up.

### Patient positioning and image acquisition

Patients underwent both CT and MRI in radiotherapy supine position prior to surgery. On CT, they were positioned with arms in abduction and hands above the head at 10° inclination and with the use of a knee support (C-Qual, CIVCO medical solutions, Reeuwijk, The Netherlands). If palpable, the tumor was marked on the skin with a CT/MRI compatible wire. CE-CT images were obtained at 3 mm slice thickness and a minimal in-plane resolution of 1 × 1 mm^2^ (Brilliance, Philips Medical Systems, Best, The Netherlands), with a delay time of 120 s after intravenous contrast agent injection (Ultravist, 80 ml, 3 ml/s) [11]. Delay time was shortened to 80s after the 6th patient according to Kuroki-Suzuki et al. in attempt to improve tumor enhancement [26].

**Figure 1:**
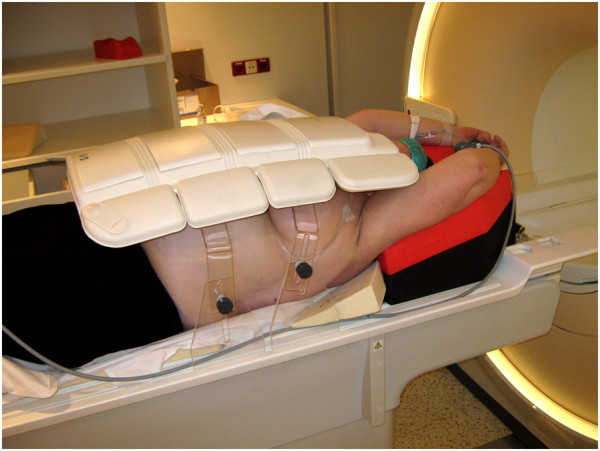
MRI patient setup in radiotherapy supine position.

For MRI, patients were positioned on an MRI compatible 10° wedge board (Thorawedge, CIVCO medical solutions, Reeuwijk, The Netherlands). To acquire MR images, an anterior receive coil was used. To prevent breast deformation by the anterior receive coil, a polymethyl methacrylate (PMMA) support was designed, which is adjustable to patient habitus and breast size. MRI patient setup is shown in Figure 1. The bore of a standard MRI scanner is too narrow to acquire images in this position. Therefore, we used a wide bore (70 cm) MRI scanner (Ingenia 1.5 T, Philips Medical Systems, Best, The Netherlands). The following 3D high resolution images were acquired: T1 weighted (T1w) fast field echo (FFE) ± fat suppression (Dixon), T2 weighted (T2w) turbo spin echo (TSE) + fat suppression, and a dynamic series of contrast-enhanced T1w images ± fat suppression after contrast agent administration. For T1w Dixon FFE MRI, acquired 3D resolution was 0.99 × 1.05 × 2.19 mm^3^ reconstructed to 0.95 × 0.95 × 1.1 mm^3^ using overcontiguous slices and for T2w TSE MRI, the voxels measured 0.78 × 0.78 × 1.2 mm^3^ acquired with a resolution of 1.25 × 1.32 × 2.41 mm^3^. In the dynamic T1w series, the first 3D image was acquired before and 6 images after intravenous contrast injection (Gadobutrol (Gadovist, Bayer), 0.1 mmol/kg, 1 ml/s), at 60s intervals with an acquired resolution of 1.20 × 1.21 × 2.41 mm^3^ reconstructed to 1.16 × 1.16 × 1.2 mm^3^ using overcontiguous slices. The total acquisition time of this protocol was 21 minutes. Small displacements between sequences during image acquisition caused by patient motion were corrected for by using a rigid mutual information registration on a box around the tumor. No breast deformation by the anterior receive coil was observed.

To quantify differences in tumor visualization on CT and MRI, the shape (1–round, 2–oval, 3–lobular, 4–irregular) and margin (1–smooth, 2–irregular, or 3–spiculated) of the tumor were rated by an experienced breast radiologist [27].

### Target volume delineation

Four experienced breast radiation oncologists independently delineated the GTV on both CT and MRI data, with at least a 4-week interval between delineation sessions, using an in-house developed software tool (Volumetool) [28]. Written delineation instructions were formulated in a consensus meeting with all observers, supervised by an experienced breast radiologist. MRI delineations were performed on preoperative 3D CE T1w images with an individually prescribed fixed window and level as determined by an experienced breast radiologist. Observers were allowed to consult other sequences, which were registered to the CE-MRI series to differentiate between structures, i.e. tumor (gadolinium uptake causes a high signal on CE T1w images), post-biopsy hematoma (blood causes a high signal on both CE and non-CE T1w images), and cysts (fluid causes a high signal on T2w images). Clinical target volumes (CTVs) were created by adding a 1.5 cm margin around the GTV, restricted by the chest wall and a 5 mm margin beneath the skin surface. Delineation of a preoperative GTV different from the tumor location as confirmed during histopathological examination of the lumpectomy specimen (gold standard) was considered as ‘misdelineation’.

### Data analysis

The conformity index (CI) and distance between the centers of mass (dCOM) for both the GTV and CTV contours as delineated by the 4 observers were calculated for all possible observer pairs. The CI per observer pair was calculated by using the following formula: $\mathrm{CI}=\frac{\text{volume}\;\text{of}\;\mathrm{agreement}}{\text{total}\;\text{encompassing}\;\text{volume}}$. Consequently, CI = 1 implies a perfect agreement among observers, while CI = 0 means there is no overlap. For dCOM, a value of 0 means that two delineations are centered at the same position.

Median values and accompanying ranges were used to describe the data since not all variables were normally distributed. A Wilcoxon signed-rank test was performed to compare paired variables using IBM SPSS Statistics 20 (Chicago, IL, USA) with a significance level of α = 0.05.

## Results

### Patients

**Figure 2:**
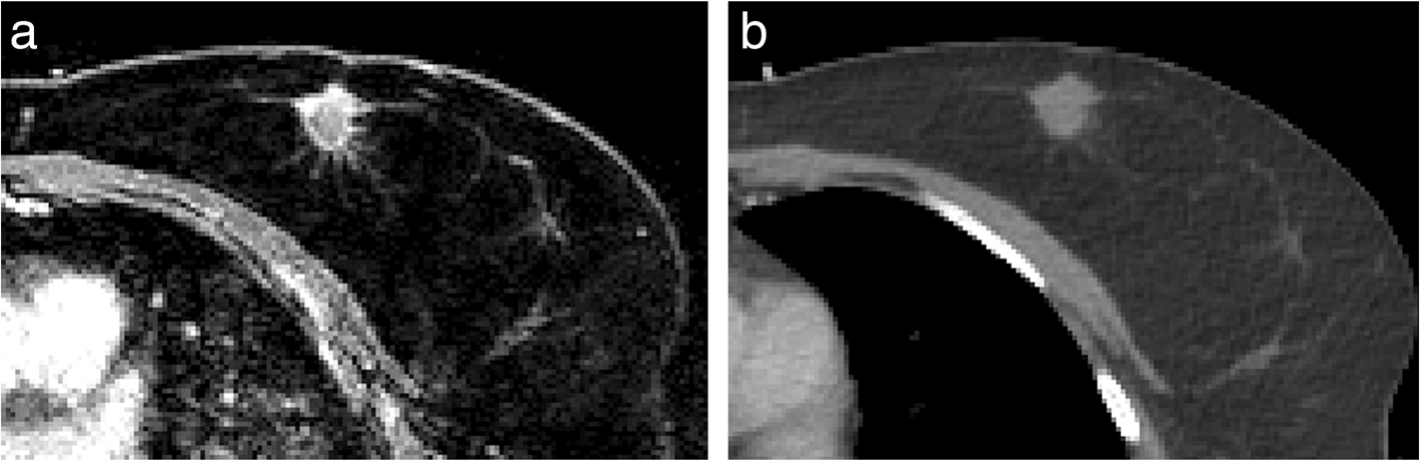
**Small peripheral branches in the transversal plane. (a)** CE-MRI and **(b)** CE-CT.

Patient and tumor characteristics are shown in Table 1. Median age was 61 years (range 48–70). Median clinical tumor diameter (as measured on diagnostic ultrasound/MRI) was 15 mm (range 7–30 mm), and median microscopic tumor diameter (as measured by histopathological examination) was 12 mm (range 6–29 mm). On CE-MRI, tumor margins were scored more spiculated compared to CE-CT (Table 1, Figure 2). Tumor shape was mainly scored as an irregular mass on CE-MRI and as a lobular mass on CE-CT.

**Table 1:** Patient and tumor characteristics

**Characteristic**	**Value**
Age (years)	48-70
Median	61
Side	
Left	7
Right	7
Histology	
Ductal carcinoma	8
Ductulolobular carcinoma	5
Tubular carcinoma	1
Clinical tumor diameter (mm)	7-30
Median	15
Microscopic tumor diameter (mm)	6-29
Median	12
Tumor visualization score on CT (median)	
Margin	2
Shape	3
Tumor visualization score on MRI (median)	
Margin	3
Shape	4

Tumor visualization scores:

Margin: 1 – smooth, 2 – irregular, or 3 – spiculated.

Shape: 1 – round, 2 – oval, 3 – lobular, 4 – irregular.

### Interobserver variability and volumes

**Figure 3:**
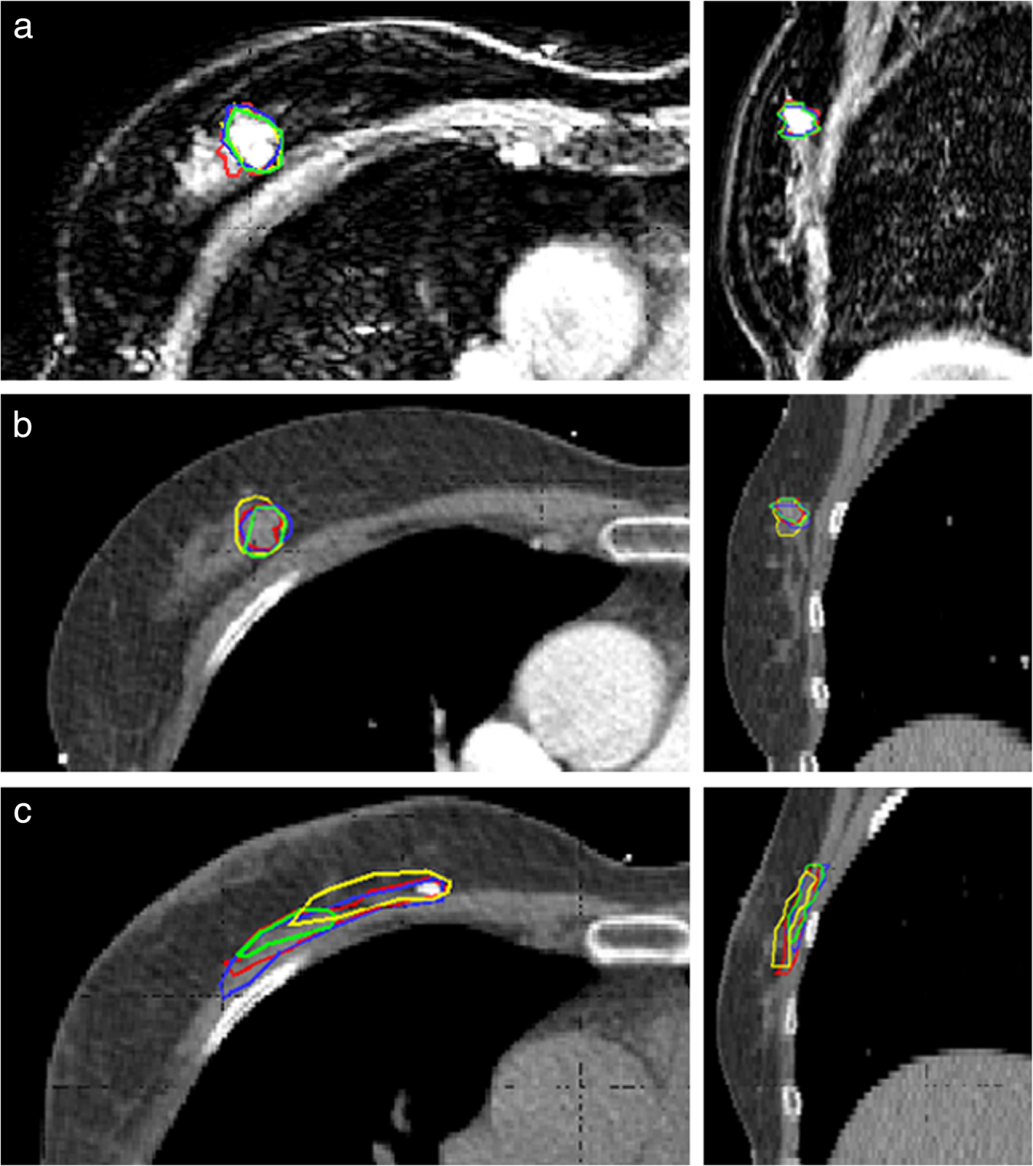
**3D GTV delineations of 4 different observers in the transversal and sagittal plane in one patient on. (a)** Preoperative CE-MRI **(b)** Preoperative CE-CT **(c)** Clinical postoperative CT.

In Figure 3a and 3b, GTV delineations of the 4 observers are shown on both preoperative CE-CT and CE-MRI in one patient. To illustrate the comparison with the current standard CT delineations after lumpectomy, postoperative delineations of this patient are shown in Figure 3c as a clinical example.

Preoperative tumor delineation resulted in a high median CI of the CTV, for both CT (0.80) and MRI (0.84)). However, the tumor was missed on CT in 2/14 patients (14%). This resulted in wide ranges in CI on CT (range 0.00-0.93 for the CTV) compared to MRI (range 0.47-0.93). The first patient in which misdelineation occurred was a patient with multiple macrocalcifications in the breast, as seen on mammography. On CE-MRI all 4 observers contoured the tumor. On CE-CT a benign lesion was contoured by 3 observers, resulting in a CI ranging from 0.00 to 0.52. The second patient had a tumor centrally located in the breast. On CE-MRI all observers contoured the tumor, while on CE-CT one observer contoured dense fibroglandular tissue resulting in a range in CI of 0.00-0.59. Outcomes of the analysis including misdelineations are provided as Additional file 1.

To only focus on differences in contouring the actual tumor, and not on tumor detection, the 2 misdelineations were excluded from further IOV and volume analysis. Results of this analysis are shown in Table 2. The CI for the GTV was significantly higher on MRI (p < 0.001) compared to CT. No difference in CI for the CTV was found (p = 0.123). Delineated volumes were significantly larger on MRI for both the GTV and CTV (both p < 0.001). There was no difference in dCOM between CT and MRI for both the GTV and CTV.

**Table 2:** Parameters of interobserver variability (misdelineations excluded from analysis)

	**CT**		**MRI**		
	**Median**	**Range**	**Median**	**Range**	**p-value**
**Mean volume (cm**^**3**^**)**					
GTV	2.1	0.3 – 21.3	2.7	0.4 – 19.4	<0.001
CTV	48.1	27.7 – 137.3	59.0	30.4 – 153.1	<0.001
**Conformity index**					
GTV	0.56	0.11 – 0.83	0.61	0.37 – 0.78	<0.001
CTV	0.82	0.39 – 0.93	0.84	0.47 – 0.93	0.123
**Mean dCOM (mm)**					
GTV	1.1	0.3 – 12.8	1.2	0.3 – 3.6	0.245
CTV	1.4	0.3 – 13.6	1.8	0.1 – 4.8	0.836

CI Conformity Index, GTV gross tumor volume, CTV clinical target volume, dCOM center of mass distance.

## Discussion

To our knowledge, this is the first study in which the feasibility of 3D CE-MRI of patients in radiotherapy supine position using a wide bore MRI scanner has been demonstrated. Different sequences of high resolution 3D CE and non-CE images were acquired with isotropic voxel sizes ≤ 1.2 mm.

In the present study, target volume delineation before lumpectomy resulted in a high agreement and small treatment volumes among observers compared to standard postoperative TB delineation as reported in literature (Table 3).

**Table 3:** Studies reporting the interobserver variability in TB, GTV, CTV and PTV delineation after breast-conserving surgery

		**Number of patients**	**Number of observers**	**Studied target volume**	**Volume (cc)¶**	**Method**	**Outcome¶**
a) CT	Struikmans 2005 [4]	18	5	TB	Mean 20 (6.4-75.8)	CI_pairs_	Mean 0.56 (0.39-0.74)
	Petersen 2007 [5]	30	3	TB	Mean 48.7 (10.3-189.3)	CI_common_	Mean 0.61 (0.27-0.84)
						dCOM	0.5-1.1^*^ (SD 0. 5–1.8)
	Hurkmans 2009 [6]	10	4	TB	40	CI_common_	Mean 0.31 (range 0.11 – 0.52)
	Coles 2009 [7]	12	2	TB	29.0 (SD 28.5)	dCOM	Median 3 (range 1–9)
					18.7 (8–116)		
	Jolicoeur 2011 [8]	66	3	TB	13.7	CI_common_	Mean 0.66
	Giezen 2012 [9]	15	4	TB	27 (SD 25)	CI_gen_	0.52 (SD 0.21)
						dCOM	4 (SD 3)
	Van Mourik 2010 [10]	8	13	CTV	n.a.	CI_pairs_	0.61^#^ (range 0.35 – 0.79)
						dCOM	7.25 (2.73 – 26.87)
	Boersma 2012 [11]	26	5	CTV	41.9 (SD 34)	CI_pairs_	0.36 (SD 0.21)
						dCOM	11 (SD 8)
	Van den Assem 2012 [12]	19	3	CTV	37.6	CI_pairs_	Mean 0.59
						dCOM	Mean 5.5
	Landis 2007 [13]	29	4	PTV	202 (65–492)	CI_pairs_	0.76 (0.52-0.92)
						dCOM	2.5 (0. 7–11.5)
b) MRI	Jolicoeur 2011 [8]	66	3	TB	10.1	CI_common_	Mean 0.96
	Giezen 2012 [9]	15	4	TB	27 (SD 26)	CI_gen_	0.32 (0.25)
						dCOM	11(SD 10)
c) CT + additional preop CT	Boersma 2012 [11]	26	5	CTV	36 (SD 31)	CI_pairs_	0.36 (SD 0.19)
						dCOM	10 (SD 7)
	Van den Assem 2012 [12]	19	3	CTV	34.7	CI_pairs_	Mean 0.68
						dCOM	Mean 2.9
d) Preop CT	Boersma 2012 [11] + vd Leij 2012 [18] (same dataset)	26	5	GTV	0.99	CI_pairs_	0.45 (SD 0.22)
						dCOM	4.3 (SD 7.6)
				CTV	37.5	CI_pairs_	Mean 0.77
						dCOM	Mean 4.4
	This study (excluding 2 misdelineations)	12	4	GTV	2.1	CI_pairs_	0.56 (0.11 – 0.83)
						dCOM	1.1 (0.3 – 12.8)
				CTV	48.1	CIpairs	0.82 (0.39 – 0.93)
							1.4 (0.3 – 13.6)
e) Preop MRI	This study (excluding 2 misdelineations)	12	4	GTV	2.7	CI_pairs_	0.61 0.37 – 0.78)
						dCOM	1.2 (0.3 – 3.6)
				CTV	59.0	CI_pairs_	0.84 (0.47 – 0.93)
						dCOM	1.8 (0.1 – 4.8)

*Range of the mean dCOM in x-y and z-direction and range in SD.

^#^CI values of one observer with all twelve observers were averaged.

^¶^Volume, CI and dCOM: reflected in median (range) or mean ± SD, unless stated differently.

TB Tumor Bed volume, GTV Gross Tumor Volume, CTV Clinical Target Volume, PTV Planning Target Volume.

CI Conformity Index, method of analysis (common, pairs, general) as described by Van Kouwenhoven et al. [27].

dCOM Distance between the Centers of Mass (mm).

Since the optimal imaging modality for preoperative target volume delineation was unknown, delineation was studied on both CT and MRI. MRI appeared to be essential for tumor detection. For tumor delineation itself, the CI of the GTV was significantly higher on MRI and ranges on CT were wider. However, median differences were small (0.05) and may not be considered clinically relevant. For the CTV, no significant difference was found, since interobserver differences are blurred when expanding structures while uniformly excluding the skin and chest wall. However, more tumor spiculations and irregularities were observed on MRI due to its high spatial resolution (Table 1, Figure 2). This did not appear to result in a decreased GTV conformity on MRI compared to CT.

The more irregular and spiculated tumor visualization on CE-MRI might have caused the significantly larger target volumes on MRI. Thin branches in the cranio-caudal or medio-lateral direction caused a relatively large volume expansion when applying a CTV margin. Even though large volumes can lead to increased toxicity and worse cosmesis, these effects do not outweigh the chances of not including peripheral tumor branches in the target volume, especially in APBI. However, despite the high level of consensus among observers, we acknowledge that no definitive statements can be made about the accuracy of the delineations, with the lack of pathologic validation of these branches as the gold standard. A pathology study must validate whether these branches are actual tumoral extensions or rather fibrotic strands or interstitial reactions, before standard inclusion of these branches in the preoperative GTV. With the implementation of high resolution imaging, the strict boundary between the GTV and the CTV with its microscopic spread might be fading. The appropriate preoperative CTV margin on MRI is therefore subject to debate and will also be further refined according to the future information about the appearance of local recurrences in the breast in the APBI studies [22].

In the last decade, several other attempts have been made to improve the current postoperative target volume delineation (Table 3b and c). Delineation on postoperative MRI resulted in contradicting results [8,9]. Jolicoeur et al. found an improved IOV and smaller volumes, while Giezen et al. found similar volumes with a degraded IOV. In two other studies, IOV was assessed on postoperative CT while preoperative CE-CT images in the same treatment position were provided [11,12]. This resulted in an improved IOV in one of these studies. Preoperative delineation was studied on CE-CT by Boersma et al., resulting in a low IOV, which was in line with our study findings (Table 3d) [11].

Our reported findings on preoperative MRI-guided delineation resulted in high and stable conformity among observers (Table 3e). Furthermore, our preoperatively delineated GTVs were considerably smaller compared to postoperative volumes reported in literature (Table 3a). CTVs were larger, although preoperative volumes would have less outliers since there is no seroma formation. The larger CTVs in our study were caused by a uniform 1.5 cm volume expansion, while the postoperative results in Table 3a reflect ‘boost’ volumes, in which the microscopic resection margin is often subtracted from this margin. PTVs were not compared in this study, since PTV margins are institution-dependent due to the method of position verification being practiced. These PTV margins might even be changed or improved in a preoperative setting, due to less volume distortions. Overall, the high CI in combination with the small and stable volumes in this study, imply that a future neoadjuvant irradiation would be more accurate and lead to less toxicity.

When comparing our results to published data in Table 3, we have to be aware of the different methods used in the other studies. For instance, the method of CI calculation, observer backgrounds and multi-centricity of a study can influence the observed results regarding IOV [29]. Interobserver studies often use small patient groups due to the high workload (Table 3). Furthermore, it has to be noted that the CI is volume dependent. The smaller the volume being studied, the more the CI is influenced by small interobserver differences. This especially accounts for our small preoperative GTVs, but also emphasizes that when comparing different studies, the studied volume (i.e. GTV, TB, CTV or PTV) must be taken into account.

Can we, from the results of this study, conclude that MRI superior to CT for preoperative tumor delineation? In this study, MRI was essential for tumor detection. However, alternatives for tumor detection can be considered, e.g. optimizing CT parameters like contrast-enhancement of the tumor, or clearly marking the tumor by fiducials. This might be easier to implement, less time consuming and less expensive. When using preoperative imaging for a preoperative irradiation or ablative interventional technique, treating another area but the GTV would be unacceptable. Furthermore, more detail could be visualized by MRI, which could contribute to an accurate target definition. Therefore, in our future studies, CE-MRI in radiotherapy supine position will be used in addition to CT, as CT is required for treatment planning. In our institute, an MRI linear accelerator is being developed in collaboration with Philips Medical Systems (Best, The Netherlands) and Elekta (Stockholm, Sweden) [30]. This system can provide online tumor tracking using MRI during radiotherapy, which makes it possible to adapt the plan to the actual tumor position. The results of our study show that a preoperative irradiation of breast tumors could be beneficial in terms of delineation consistency and treatment volumes. There would be more certainty that the correct target is delineated when the tumor is in situ. Moreover, preoperative target volumes would probably be more stable in the absence of seroma formation, and not being subject to seroma shrinkage [17,31]. The advantages of preoperative CE-MRI for treatment planning will be further studied with respect to the dosimetric consequences [32]. CE-MRI in supine position could also be used for other purposes. For instance, it might provide additional information to improve the consistency in target volume definition in standard postoperative CT-guided delineation [11]. Furthermore, it might aid tumor localization for breast conserving surgery or interventional procedures [33].

## Conclusions

In conclusion, preoperative target volume delineation resulted in small treatment volumes with a high consistency among observers. MRI appeared to be necessary for tumor detection and the visualization of irregularities and spiculations. Regarding delineation of the tumor itself, no clinically relevant differences in interobserver variability among imaging modalities were observed. These results will be used to study the potential for a future MRI-guided and neoadjuvant radiotherapy.

## Abbreviations

CE: Contrast-enhanced; CI: Conformity index; CT: Computed tomography; CTV: Clinical target volume; dCOM: Distance between centers of mass; FFE: Fast field echo; GTV: Gross tumor volume; IOV: Interobserver variability; MRI: Magnetic resonance imaging; PTV: Planning target volume; TB: Tumor bed; T1w: T1 weighted MRI sequence; T2w: T2 weighted MRI sequence; TSE: Turbo spin echo.

## Competing interests

The authors declare that they have no competing interests.

## Authors’ contributions

All authors read and approved the final manuscript. MD participated in the design of the study, study coordination, data acquisition, data analysis, drafting the manuscript. MP participated in the design of the study, acquisition of the data, revised the manuscript critically for intellectual content. ID participated in acquisition of the data and revised the manuscript critically for intellectual content. CK participated in acquisition of the data and revised the manuscript critically for intellectual content. RT participated in acquisition of the data and revised the manuscript critically for intellectual content. AK participated in data analysis and interpretation, revised the manuscript critically for intellectual content. HV participated in the design of the study, interpretation of the data and revised the manuscript critically for intellectual content. MB participated in the design of the study and revised the manuscript critically for intellectual content. MV participated in the design of the study and revised the manuscript critically for intellectual content. BA participated in the design of the study, acquisition of the data, helped to draft the manuscript and revised it critically for intellectual content. DVB participated in the design of the study, acquisition and interpretation of the data, helped to draft the manuscript and revised it critically for intellectual content.

## Supplementary Material

Additional file 1 **Parameters of interobserver variability including misdelineations on CT, which resulted in CIs of 0.00 and high dCOMs.** In the manuscript, these outliers were excluded from analysis and shown in Table 2. Click here for file
