# Supplementary material for: MRI and CT imaging for preoperative target volume delineation in breast-conserving therapy
Source: Radiat Oncol. 2014 Feb 26;9:63. doi: 10.1186/1748-717X-9-63 (PMC3942765; doi:10.1186/1748-717X-9-63)
Supplement: Additional file 1 — Parameters of interobserver variability including misdelineations on CT, which resulted in CIs of 0.00 and high dCOMs. In the manuscript, these outliers were excluded from analysis and shown in Table 2. [file 1748-717X-9-63-S1.doc]

| **Additional file 1**  Parameters of interobserver variability including misdelineations on CT, which resulted in CIs of 0.00 and high dCOMs. In the manuscript, these outliers were excluded from analysis and shown in Table 2. | | | | | |
| --- | --- | --- | --- | --- | --- |
|  | **CT** | | **MRI** | |  |
|  | Median | Range | Median | Range | p-value |
| **Mean volume (cm^3^)**  GTV  CTV | 2.2  48.8 | 0.3 – 21.3  27.7 – 168.4 | 2.6  59.0 | 0.4 – 21.3  30.4 – 153.1 | 0.009  <0.001 |
| **Conformity index**  GTV  CTV | 0.54  0.80 | 0.00 – 0.83  0.00 – 0.93 | 0.60  0.84 | 0.37 – 0.78  0.47 – 0.93 | <0.001  0.003 |
| **Mean dCOM (mm)**  GTV  CTV | 1.3  1.6 | 0.3 – 77.9  0.3 – 77.6 | 1.2  1.7 | 0.2 – 3.6  0.1 – 4.8 | 0.004  0.172 |
| CI Conformity Index, GTV gross tumor volume, CTV clinical target volume, dCOM center of mass distance. | | | | | |
